# Supplementary material for: Deprescribing as an Opportunity to Facilitate Patient-Centered Care: A Qualitative Study of General Practitioners and Pharmacists in Japan
Source: Int J Environ Res Public Health. 2023 Feb 17;20(4):3543. doi: 10.3390/ijerph20043543 (PMC9962748; doi:10.3390/ijerph20043543)
Supplement: Supplementary file 1 [file ijerph-20-03543-s001.zip › ijerph-2157090-supplementary.pdf]

Table S1. COREQ-32 checklist

No	Item	Guide questions/description
**Domain 1: Research team and reflexivity**		
Personal Characteristics		
1	Interviewer/facilitator	1) Kenya Ie (KI), 2) Reiko Machino (RM)
2	Credentials	1) MD, MPH, PhD, 2) PharmD
3	Occupation	1) Physician (General Practice), 2) Research assistant
4	Gender	1) Male, 2) Female
5	Experience and training	1) Clinical experience in general practice and completed training in qualitative study through his MPH and PhD training.<br>2) Clinical experience in hospital and community pharmacy and on-the-job qualitative research training
Relationship with participants		
6	Relationship established	Participants at two study facilities had established relationships with KI through collaboration in clinical practice.
7	Participant knowledge of the interviewer	Some participants knew the interviewer prior to the interview.
8	Interviewer characteristics	KI: Served as a member of the deprescribing team at a hospital.
**Domain 2: study design**		
Theoretical framework		
9	Methodological orientation and Theory	Thematic analysis / Theory of planned behavior
Participant selection		
10	Sampling	Purposeful sampling
11	Method of approach	Face-to-face and e-mail
12	Sample size	35
13	Non-participation	N/A
Setting		
14	Setting of data collection	Two community hospitals, four primary care clinics, and a community pharmacy.
15	Presence of non-participants	A co-investigator presented at the group interview at one of FGIs
16	Description of sample	Described in the Results section
Data collection		
17	Interview guide	Described in the Methods section
18	Repeat interviews	N/A
19	Audio/visual recording	Audio recording was used.
20	Field notes	KI and RM made field notes during interviews when necessary
21	Duration	45-60 minutes
22	Data saturation	Yes. Researchers confirmed data saturation when the themes were repeated among multiple transcripts.
23	Transcripts returned	No
**Domain 3: analysis and findings**		

Data analysis		
24	Number of data coders	Two (KI and RM)
25	Description of the coding tree	Hierarchical coding tree with main and sub themes.
26	Derivation of themes	Themes were derived from data obtained through the focus-group interview. Simultaneously, the theory of planned behavior was to capture emergent themes in the transcript.
27	Software	N/A
28	Participant checking	No. Participants were informed that they had chance to ask questions and to give feedback when necessary but none asked to review the data.
Reporting		
29	Quotations presented	Yes
30	Data and findings consistent	Yes
31	Clarity of major themes	Yes
32	Clarity of minor themes	Yes
